# Prevalence and Molecular Characterization of *Bartonella* spp. in Ectoparasites of Cats and Dogs in Northwestern Italy

**DOI:** 10.3390/ani15162402

**Published:** 2025-08-16

**Authors:** Angela Maria Catania, Laura Tomassone, Alberto Tarducci, Elena Grego

**Affiliations:** Department of Veterinary Science, University of Turin, 10095 Grugliasco, TO, Italy; angelamaria.catania@unito.it (A.M.C.); laura.tomassone@unito.it (L.T.); alberto.tarducci@unito.it (A.T.)

**Keywords:** *Bartonella henselae*, fleas, ticks, cats, dogs, zoonotic risk

## Abstract

*Bartonella* spp. is a Gram-negative bacterium transmitted by arthropod vectors, implicated in a range of zoonotic infections affecting both humans and animals. Among zoonotic species, *B. henselae* is primarily associated with domestic cats and *B. vinsonii* with dogs. In Italy, *Bartonella* infections have been reported in both southern and northern regions. This study investigates the presence of *Bartonella* spp. in fleas and ticks collected from companion animals in Piedmont region, northwestern Italy. A total of 176 flea and 85 tick specimens were obtained from 92 animals (dogs and cats) between May 2018 and February 2020. Arthropods were morphologically identified using identification keys, and screened for *Bartonella* DNA by PCR targeting the *16s* rRNA gene. Positive samples were further analyzed by amplifying the *rpoB* gene. Selected *16s*-positive and all *rpoB*-positive samples were sequenced and subjected to phylogenetic analysis. Fleas were mostly identified as *Ctenocephalides felis*, recovered from 44 cats and 12 dogs; a single *C. canis* specimen was found in one cat. Ticks were *Ixodes ricinus* (from 14 cats and 10 dogs), *Rhipicephalus sanguineus* s.l. (3 cats, 7 dogs), and *Dermacentor marginatus* (one dog). *Bartonella* prevalence was 38.4%, with 34.2% positivity in fleas and 45.6% in ticks. All sequences corresponded to *B. henselae*. These findings confirm the active circulation of *B. henselae* in ectoparasites of pets and raise questions about the potential role of ticks in its transmission.

## Simple Summary

*Bartonella henselae* is a bacterium that can be transmitted from animals to humans, mainly through insect vectors like fleas. It is commonly found in cats, which often do not show signs of illness, but the infection can pose a risk to humans. Dogs can also carry other *Bartonella* species. In this study, we investigated the presence of *Bartonella* spp. in fleas and ticks collected from pets in Piedmont region, northwestern Italy. We analyzed 176 *Ctenocephalides* spp. fleas and 85 Ixodid ticks collected from 92 companion animals (dogs and cats), using molecular testing. We found that 38% of the arthropod samples carried *Bartonella* DNA. Further genetic analysis confirmed all positive samples as *Bartonella henselae*. These results show that *B. henselae* is actively circulating among ectoparasites that infest companion animals in this region. The presence of the bacterium in both fleas and ticks suggests that ticks may also play a role in its transmission. Regular use of parasite control treatments in pets is essential, and veterinarians play a key role in preventing zoonotic risks and promoting public health.

## 1. Introduction

Fleas and ticks are hematophagous ectoparasites able to transmit several pathogens relevant to veterinary and public health [1]. Their ubiquitous presence and ability to transmit pathogens, including bacteria, viruses, and protozoa, underscore their critical role in the epidemiology of vector-borne diseases [1]. The burden of infestation varies geographically, but in the regions of the Mediterranean Basin, these ectoparasites are widespread, due to favorable climatic conditions and host availability, and pose a continuous threat to both animal and human health [2]. Among transmitted pathogens, *Bartonella* spp. are considered emerging [3]. These Gram-negative bacteria are primarily transmitted by arthropod vectors and can infect a wide range of mammalian hosts, including humans [4]. Zoonotic *Bartonella* species include *B. bacilliformis,* which causes Oroya fever and Peruvian wart [5], *B. quintana*, associated with trench fever [6], and *B. henselae*, responsible for Cat Scratch Disease (CSD) [7].

Domestic cats (*Felis catus*) are the primary reservoir of *B. henselae* [8]. Although typically asymptomatic, some infected cats may develop transient fever, anemia, or lymphadenopathy [9]. The absence of clinical signs suggests an effective bacterial-host adaptation. However, infected cats (also asymptomatic) can harbor the bacterium in their bloodstream for extended periods, facilitating transmission via ectoparasites [10]. A significant percentage of cats, including both stray and domestic populations, test positive for *Bartonella* through blood cultures and serological tests [11]. Risk factors for infection include age, infestation by fleas, and outdoor access [11]. A meta-analysis estimated a global prevalence of *B. henselae* in cats at 15.3% [12]. Bacteremia prevalence in stray cats in northern Italy has been reported as high as 23% [13]. Seroprevalence in pet cats in Tuscany (central Italy) was reported at 16%, while in stray cats in northern Italy, it was estimated at 39% [13]. The prevalence in Italy varies across different regions, suggesting regional variations in exposure and transmission.

Dogs (*Canis familiaris*) may also carry *B. henselae* and are considered the main reservoir for *B. vinsonii berkhoffii*, which has been associated with endocarditis and other systemic diseases in both dogs and humans [14]. Prevalence of *Bartonella* infection in dogs varies widely depending on geographical location, diagnostic method, and studied population. The global prevalence of *Bartonella* spp. in dogs is estimated at around 3.6% [12]. Italy shows varying prevalence depending on the region. Serological investigations have detected the presence of antibodies against *Bartonella* spp. in 6% and 28% of dogs in Sardinia (southern Italy) and Emilia-Romagna (central Italy), respectively [13]. Clinical signs in dogs vary widely and are often not directly attributable to *Bartonella* infection [15]. However, studies suggest a link to severe conditions such as cardiac dysfunctions and cancer [16,17]. Due to their exposure to shared environments and arthropod vectors, dogs are also considered important epidemiological sentinels for human infection [18].

In humans, clinical manifestations depend on the *Bartonella* species involved and the host’s immune status. Immunocompromised individuals are at greater risk of severe complication including bacillary angiomatosis and peliosis [19,20]. CSD, caused by *B. henselae*, may cause localized lymphadenopathy, skin lesions, fatigue and muscle pain in healthy individuals. Other zoonotic *Bartonella* species, such as *B. alsatica*, *B. koehlerae*, *B. vinsonii*, *B. washoensis*, and *B. rochalimae*, have been associated with myocarditis, endomyocarditis, granulomatosis, and neuroretinitis [21,22,23,24]. Moreover, neurobartonellosis cases are emerging, and *Bartonella* species have been documented in diverse neurological conditions affecting the peripheral and central nervous systems [25].

Bartonellosis is endemic in several regions and closely linked to vector presence [3]. While fleas are recognized as vectors, the role of ticks in *Bartonella* transmission remains uncertain [26]. Although the disease is more common in warm and temperate climates, climate change is expanding its geographical range, necessitating increased vigilance and management under the One Health framework [27]. Bartonellosis and other vector-borne diseases (VBDs) have been documented in several Mediterranean countries, including Italy, with prevalence influenced by sample size, geography, and animal lifestyle [2,28,29,30,31]. However, data on the presence and genetic characterization of *Bartonella* spp. in northwestern Italy are limited. A recent study detected *B. schoenbuchensis* and Candidatus *B. gerbillinarum* in fox populations, highlighting the need for further research to clarify the epidemiology of *Bartonella* spp. and their potential interactions with domestic animals in the region [32].

By introducing ectoparasites into domestic settings, companion animals may influence the dynamics of vector-borne diseases transmission [33]. Despite the documented presence of *Bartonella* species in companion animals and the recognized endemicity of bartonellosis in Italy, there remains a significant knowledge gap regarding the current circulation and genetic characteristics of *Bartonella* species within ectoparasites (fleas and ticks) infesting companion animals in northwestern Italy.

This study aims to address this critical gap by assessing the prevalence of *Bartonella* spp. in fleas and ticks parasitizing cats and dogs in northwestern Italy and evaluate its circulation in the study area.

## 2. Materials and Methods

### 2.1. Animals and Ectoparasite Collection

Between May 2018 and February 2020, 92 companion animals (62 cats and 30 dogs) were examined during routine veterinary consultations. The study was conducted in the Piedmont region, located in northwestern Italy. This region presents a diverse landscape, characterized by the western Alps to the north and west, rolling hills, and extensive lowland plains in the central and eastern parts. This geographical variability creates a range of climatic conditions and natural habitats; from mountainous and forested areas providing suitable environments for ticks, to more temperate plains potentially favoring fleas. This mixed environment contributes to varied exposure risks for companion animals to different ectoparasite species, depending on their lifestyle and access to outdoor environments. Ectoparasites were collected by veterinary practitioners using entomological forceps, stored in labeled 1.5 mL microtubes containing 70% ethanol, and kept at room temperature until processing.

A questionnaire was completed for each animal to record sex, breed, health status, presence of ectoparasite, outdoor access, and cohabitation with other animals.

A clinical case was investigated involving a 2-year-old female Border Collie whose ectoparasites (ticks) tested positive for *Bartonella henselae* during a routine screening. Six months later, the dog developed clinical signs consistent with splenic disease, prompting a splenectomy. The excised splenic tissue was submitted to an external diagnostic service for histopathological evaluation. In parallel, DNA was extracted from the tissue and subjected to PCR analysis targeting the same genes (as described below) to assess the infection and the potential tissue involvement.

### 2.2. Characterization and Detection of Bartonella *spp*.

Ectoparasites were examined under a stereomicroscope and identified at the species and stage level using morphological identification keys [34,35].

Tick and flea DNA were extracted either individually or from pools composed of 2–6 specimens of the same species collected from the same host. Pooling was performed only when multiple specimens of a single species were available from an individual animal, to balance sensitivity and sample throughput. A total of 261 individual ectoparasites (176 fleas and 85 ticks) were collected from the animals; these were processed into 125 distinct molecular samples (62 pooled and 63 individual samples) for *Bartonella* screening. Specifically, of the 176 fleas, 140 were pooled and 37 were tested individually. Of the 85 ticks, 59 were pooled and 26 were tested individually. Specimens were rinsed with sterile water, air-dried, cut with a sterile blade and mechanically homogenized using sterile pestles. The spleen tissue from the Border Collie dog (10 mg) was cut in small pieces with a sterile blade. The homogenates were incubated overnight at 56 °C with proteinase K solution. Total nucleic acids were extracted using the DNAzol™ reagent (Thermo Fisher Scientific, Waltham, MA, USA) following the manufacturer’s instructions. DNA concentration and purity were assessed using a NanoDrop ND 2000 spectrophotometer (Thermo Fisher Scientific, Waltham, MA, USA).

PCR was performed to amplify a 296 bp fragment of the *16s* rRNA gene, using the primers 5F 5′-YCTTCGTTTCTCTTTCTTCA-3′ and 5′-AACCAACTGAGCTACAAGCC-3′ [36]. PCR reactions were performed in a 25 μL volume contained in a 1X MyTaq Red Reaction Buffer, 200 nM of each primer, 5 U MyTaq Red DNA Polymerase (Bioline-Meridian Bioscience Inc., Taunton, MA, USA), and 1 μL DNA (50–100 ng). The PCR cycling conditions were initial denaturation at 95 °C for 1 min, followed by 40 cycles of 95 °C for 30 s, annealing at 55 °C for 30 s, extension at 72 °C for 30 s, and a final extension at 72 °C for 10 min.

Samples testing positive were further subjected to PCR amplification targeting an 825 bp fragment of the *rpoB* gene using the primers 1400F 5′-CGCATTGGCTTACTTCGTATG-3′ and 2300R 5′-GTAGACTGATTAGAACGCTG-3′ [37]. The PCR mixture and conditions were the same as above, except the extension step, which was 45 s, and the final extension, which was 5 min at 72 °C.

PCR products were visualized on a 2% agarose gel stained with SYBR Safe (Thermo Fisher Scientific, Waltham, MA, USA), purified using the ExoSAP-IT™ Product Clean-up kit (GE Healthcare Limited, Chalfont, UK) and sequenced via an external service (BMR Genomics, Padua, Italy).

### 2.3. Phylogenetic Analysis

Sequence identity was confirmed via BLASTn searches against the NCBI GenBank database [38].

Sequence editing, analysis and similarity comparison calculations between sequences were performed using BioEdit software version 7.0.9 [39].

For phylogenetic inference, representative *Bartonella* spp. sequences were selected from GenBank based on geographic relevance and gene completeness. Sequences were aligned using Clustal X v2.1 [40] present in BioEdit v7.0.9 software [39]

Bayesian phylogenetic trees were constructed using MrBayes v3.2.7a [41], with Markov Chain Monte Carlo (MCMC) analysis run for 100 million generations, sampling every 1000 generations, and discarding the first 25% as burn-in. Resulting trees were visualized and edited using FigTree v1.4.4.

### 2.4. Statistical Analysis

Data analysis was performed using R software (https://www.R-project.org/) [42]. The possible association between animals’ characteristics and ectoparasite infestation or *Bartonella* infection were assessed with the Chi-square test. A *p*-value < 0.05 was considered statistically significant. Prevalence estimates for arthropod infestation and *Bartonella* spp. infection were calculated with 95% confidence intervals (95% CI).

## 3. Results

The details of the animals involved in this study are summarized in Table 1.

Out of 62 cats, 45 (72.6%, 95% CI: 59.8–83.1) were infested with fleas and 17 (27.4%, 95% CI: 16.9–40.2) with ticks. Among the 30 dogs, 12 (40.0%, 95% CI: 22.6–59.4) were infested by fleas and 18 (60.0%, 95% CI: 40.6–77.3) by ticks. Cats were significantly more infested with fleas (*p* < 0.05), whereas dogs with ticks (*p* < 0.05). Purebred dogs showed a significantly higher tick infestation rate than mixed breeds (*p* < 0.01).

No significant associations were observed between ectoparasite infestation and sex, ownership status, cohabitation, or outdoor access (*p* > 0.05).

A total of 176 fleas and 85 ticks were collected from the sampled animals. *Ctenocephalides felis* was the predominant flea species (n = 175), while a single *Ctenocephalides canis* specimen was identified. *C. felis* was recovered from 44 cats and 12 dogs, while *C. canis* was found on one cat (Table 2).

Ticks were identified as *Rhipicephalus sanguineus* sensu lato (n = 46; 43 adults, 3 nymphs), *Ixodes ricinus* (n = 38; 26 adults, 12 nymphs), and *Dermacentor marginatus* (n = 1 adult). *R. sanguineus* was found on 10 animals (3 cats, 7 dogs), *I. ricinus* on 24 animals (14 cats, 10 dogs), and *D. marginatus* on a single dog (Table 2).

No significant differences in infestation rates were found between animals living alone and those in contact with other animals (*p* > 0.05). Similarly, infestation rates did not significantly differ between healthy and diseased animals or between indoor and outdoor animals (*p* > 0.05).

Of the 125 ectoparasite samples tested (62 pooled and 63 single), 48 (38.4%, 95% CI: 29.8–47.5) were positive for the *Bartonella 16s* rRNA gene. These included 27 flea samples (34.2%, 95% CI: 23.9–45.7) and 21 tick samples (45.6%, 95% CI: 30.9–61.0). Positive samples were obtained from 28 animals (15 cats and 13 dogs).

All *16s*-positive samples were subjected to confirmatory PCR targeting the *rpoB* gene, of which six (three fleas and three ticks) tested positive.

As regards the clinical case, the Border Collie was presented at the veterinary clinic with abdominal discomfort and ultrasound evidence of splenomegaly six months after tick collection. Splenectomy was performed and histological examinations revealed diffuse hemorrhagic necrotizing splenitis. PCR on splenic tissue was positive for *B. henselae*. Post-operative recovery was completed; no evidence of neoplasia was found.

**Figure 1:**
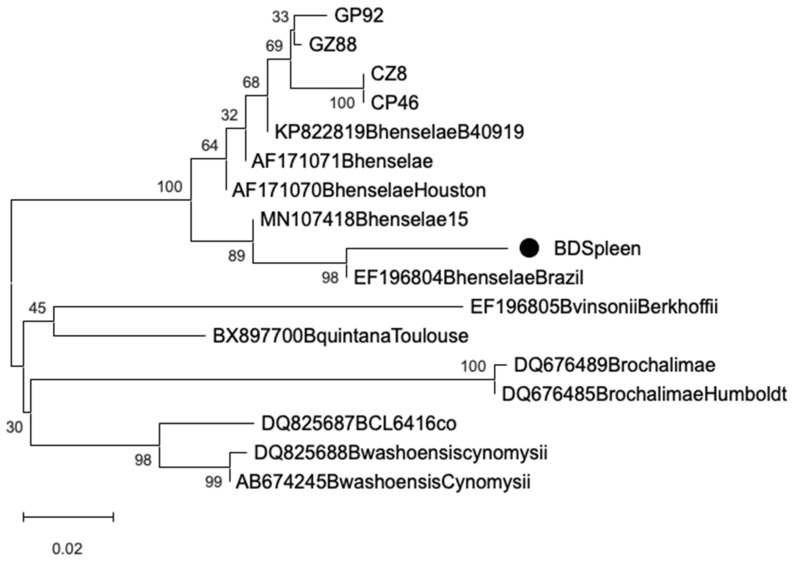
Bayesian phylogenetic tree of *Bartonella* spp. *rpoB* gene from ectoparasites (GP: cat–flea; GZ: cat–tick; CP: dog–flea; CZ: dog–tick; BD: splenic tissue samples), and reference strains retrieved from GenBank. Markov Chain Monte Carlo (MCMC) 100 sampling every 100,000 generations. The scale bar represents 0.02 nucleotide substitutions per site.

Sequencing of the *16s* rRNA amplicons showed 97.3–99% identity with *Bartonella henselae* (GenBank Accession No.: MT095053.1). Phylogenetic analysis of the *rpoB* gene sequences confirmed clustering with reference *B. henselae* strains. All sequences clustered within a single, well-supported *B. henselae* clade, with no evidence of additional *Bartonella* species. Sequences from cat- and dog-associated fleas and ticks grouped closely, showing no host- or vector-specific divergence. Notably, the splenic sample clustered with a human-derived *B. henselae* isolate (Figure 1).

## 4. Discussion

This study investigated the presence of *Bartonella* spp. in fleas and ticks collected from companion animals in the Piedmont region, northwestern Italy.

The infestation patterns detected in our animal sample likely reflected species–specific behavior and vector ecology: dogs were more frequently infested with ticks, while cats showed a higher prevalence of flea infestation [1]. Among the tick species identified, *R. sanguineus* s.l. was the most common, consistent with previous reports from Italy [43]. Other identified species included *Ixodes ricinus* and *Dermacentor marginatus*, which are typically associated with wooded habitats and are less commonly infesting domestic dogs in urban areas. Nearly all fleas were identified as *C. felis*, predominantly found on cats, consistent with previous studies conducted both in Italy and in other countries [44,45,46]. The wider distribution of *C. felis* compared to *C. canis* is well documented [47]. Both flea species can infest multiple hosts, including humans, particularly during warm months [48].

In terms of risk factors, purebred dogs exhibited a significantly higher burden of tick infestation compared to mixed-breed dogs. However, no significant difference in ectoparasite infestation was observed in relation to ownership status (owned vs. shelter animals), outdoor access, or contact with other animals. Similarly, the detection of *Bartonella* DNA did not significantly differ between dogs and cats or between subgroups based on living conditions. While cats were more frequently infested with fleas, this did not translate into a significant difference in *Bartonella* prevalence. The heterogeneity of our animal sample could justify these results; further studies, performed on a higher number of pets and with a more focused sample selection, could help in highlighting possible risk factors for *Bartonella* infection in the study area. *Bartonella* spp. DNA was detected in 34.2% of flea samples and 45.6% of tick samples, but the only species identified by *16s* rRNA and *rpoB* gene sequencing was *B. henselae*. Previous studies reported a *B. henselae* prevalence rate of 21.4% in cats and 14.3% in fleas in Italy [30,49]. Variability in prevalence rates in the literature may reflect differences in sample types (e.g., blood vs. ectoparasites), diagnostic techniques (molecular vs. serological), host species, or animal-related factors such as ownership status and ectoparasite control practices [49,50].

The phylogenetic analysis confirmed the presence of *B. henselae* in ectoparasites collected from both cats and dogs, as well as in the splenic tissue of a clinically affected dog, supporting the notion of active circulation and potential systemic involvement in companion animals. Sequencing of the *16s* rRNA gene showed high identity (97.3–99%) with *B. henselae* reference strains, while *rpoB* gene analysis further confirmed species-level identity through clustering within a well-supported *B. henselae* clade. All sequences—whether derived from ticks, fleas, or the splenic sample—fell into a single genetic cluster, suggesting minimal variability among strains circulating in the region. No host- or vector-specific divergence was observed, with sequences from cat- and dog-associated ectoparasites grouping closely. This indicates a low degree of genetic heterogeneity in local *B. henselae* populations and supports their adaptability to multiple hosts and vectors. Of particular note, the ectoparasites (ticks) collected from the same dog that later developed clinical splenic disease also tested PCR-positive for *B. henselae*. The subsequent detection of the same bacterial species in the excised splenic tissue—confirmed through sequence identity and phylogenetic clustering—supports a potential link between vector exposure and systemic infection in this individual case. While causality cannot be definitively established, this temporal and molecular association reinforces the hypothesis of tick-mediated transmission and tissue tropism of *B. henselae* in dogs. These findings emphasize the value of integrating molecular diagnostics with clinical and epidemiological data in veterinary settings. They also highlight the need for greater awareness of *Bartonella* spp. as possible contributors to systemic disease in dogs, particularly when compatible clinical signs such as splenomegaly or vascular lesions are present. Phylogenetic tools provide a powerful means to trace infection sources and clarify host-pathogen-vector relationships, ultimately informing both diagnostics and preventive strategies within a One Health framework.

A noteworthy outcome was the high prevalence of *B. henselae* DNA in tick samples, raising attention about their potential role in the bacteria transmission cycle. While cat fleas are an established vector of *B. henselae*, the role of ticks in its transmission remains controversial [51,52,53,54]. Nevertheless, several studies have detected *B. henselae* DNA in tick species such as *R. sanguineus* and *I. ricinus* [51,55]. Reported infection prevalence varies from 0.6% in *Ixodes* spp., collected from dogs in Denmark [56], to up to around 40% in questing *I. ricinus* from Germany, France and Portugal [57]. Moreover, co-infections involving *B. henselae* and other tick-borne pathogens (e.g., *Rickettsia* spp., *Borrelia* spp., and *Anaplasma* spp.) suggest possible co-transmission [58,59]. In addition, experimental infection in *R. sanguineus* ticks confirmed the transstadial transmission of *B. henselae* larvae to nymphs and indicates the possible re-transmission of the bacteria through saliva during blood meal [60]. Human cases of *B. henselae* infection following tick bites have also been reported [53,61,62], further supporting the potential vectorial role of ticks. A study carried out in Sweden showed a higher seroprevalence against *B. henselae* and *B. quintana* in patients exposed to ticks, compared to healthy blood donors [63]. In conclusion, the possible implication of Ixodix ticks in *Bartonella* spp. transmission is supported by new evidence and deserves further investigation.

To balance detection sensitivity with sample throughput, DNA extraction was performed from both individual and pooled ectoparasite samples (up to 10 specimens of the same species and from the same host). This strategy enabled efficient detection of *Bartonella* spp. while preserving analytical feasibility. It is acknowledged that pooling may slightly reduce sensitivity when bacterial loads are low; however, it enhances the representativeness of infection at the host level and is appropriate for large-scale epidemiological screening. The use of morphologically identified pools ensured accurate species classification while maintaining the reliability of molecular results. Despite their epidemiological significance, *Bartonella* infections often remain undiagnosed in companion animals due to nonspecific or absent clinical signs. While cats are typically asymptomatic carriers, dogs may exhibit vague systemic symptoms [3]. The case of a two-year-old Border Collie with hemorrhagic splenitis and confirmed *Bartonella henselae* infection offers a compelling clinical association. This highlights the need for further investigation into the pathogenic potential of *Bartonella* spp. in companion animals. Indeed, *Bartonella* infections have been associated with both neoplastic and non-neoplastic vascular lesions in animals [17] underscoring their broader clinical relevance in veterinary medicine.

The detection of *Bartonella*-positive ectoparasites, especially from household pets, raises important public health concerns. Since pets can be reservoirs for several zoonotic agents, including *Bartonella* spp., a stronger collaboration between veterinary and medical doctors is warranted to improve awareness and communication with pet owners. Companion animals, particularly cats, are recognized reservoirs of *B. henselae*, which can be transmitted to humans primarily through skin inoculation with infected flea feces introduced during scratching or biting, Additional routes of transmission—such as mucosal exposure (oral or conjunctival), needle-stick injuries in veterinary or clinical environments, and blood transfusions—have also been documented [3,64].

A study conducted in northern Italy revealed that 82% of cats owned by patients diagnosed with cat scratch disease (CSD) tested positive for *B. henselae*, emphasizing the close link between pet infection and human disease [65]. While the incidence of reported *Bartonella*-related infections in humans in Italy remains low (<1 case per 10,000 inhabitants), this number is likely underestimated due to under-diagnosis and lack of awareness, especially among general practitioners [66]. Immunocompromised individuals are particularly at risk, as illustrated in a recent Spanish study documenting seropositivity to *Bartonella* spp. in immunosuppressed children who adopted stray cats following organ transplants [67].

Importantly, veterinarians and animal handlers are not only essential actors in disease prevention but also represent a high-risk group for occupational exposure. A study from the United States revealed the presence of *Bartonella* DNA in 28% of veterinary professionals [68], while seroprevalence among veterinary personnel in Spain reached 37.1% [69]. This significant occupational exposure underscores the need for improved biosafety protocols and regular health surveillance among veterinary workers, who may unknowingly become infected. Increased educational efforts are also needed within the veterinary community to recognize subtle or atypical signs of infection in animals, as well as to implement biosecurity measures that reduce risk of zoonotic transmission.

Our findings highlight the value of veterinarians in zoonotic surveillance and their strategic position in implementing One Health approaches to disease prevention. By identifying ectoparasite infestation and *Bartonella* positivity in companion animals, veterinarians can guide evidence-based interventions at the pet–owner interface. Animal owners should be informed about the importance of preventive care, including consistent ectoparasite control on both the host and within the household environment, especially in families with vulnerable individuals (e.g., children, the elderly, and immunocompromised patients) [70].

Despite these insights, our study had several limitations. Sampling was conducted opportunistically and depended on the availability of veterinary professionals and owner consent. This may limit the generalizability of results. Furthermore, DNA extraction was carried out from both individual and pooled ectoparasites (up to six specimens per pool from the same host and species) to optimize efficiency. While pooling may slightly reduce sensitivity in cases of low bacterial load, it increases representativeness at the host level and is a commonly accepted strategy in large-scale screening. This approach allowed us to detect *Bartonella* spp. with good sensitivity while maintaining feasibility for routine epidemiological surveillance. Morphological species identification further ensured correct classification of vectors, adding reliability to molecular findings.

## 5. Conclusions

In conclusion, the zoonotic potential of *Bartonella henselae*, confirmed by its detection in both fleas and ticks collected from companion animals, reinforces the need for integrated prevention strategies. As household pets can serve as reservoirs for *Bartonella* spp., and ectoparasites as potential vectors, awareness and cooperation between veterinarians, pet owners, and public health professionals are essential. Effective ectoparasite control is a cornerstone of disease prevention and should be applied both to animals and their shared environments.

Moreover, veterinarians have a dual responsibility: safeguarding animal health and serving as sentinels for zoonotic risks. By educating pet owners about parasite prevention, early symptom recognition, and the importance of regular veterinary check-ups, they play a crucial role in limiting transmission. Public health communication should highlight that even asymptomatic animals can carry and spread *Bartonella* spp., particularly in high-risk populations such as children, immunocompromised individuals, and professionals working closely with animals.

These findings underscore the relevance of applying a One Health approach to *Bartonella* surveillance and control. Coordinated monitoring of both vectors and hosts, coupled with molecular diagnostics, can significantly improve early detection and response strategies. Future studies should focus on defining the role of ticks in *Bartonella* transmission, exploring potential co-infections, and expanding our understanding of the clinical implications in domestic animals. This integrated perspective will be key to reducing the risk of transmission across species barriers.

## Figures and Tables

**Table 1:** Characteristics of the cats (n = 62) and dogs (n = 30) from the Piedmont region, gathered from questionnaires to pet owners during veterinary consultations. ‘N.D.’ (not determined) refers to animals for which the information was not available.

		Dogs			Cats	
	Yes	No	N.D.	Yes	No	N.D.
Female sex	15	14	1	29	27	6
Owned	25	2	3	37	19	6
Purebred	13	13	4	2	44	16
Neutered/Castrated	9	15	6	22	19	21
Cohabitation with other animals	16	8	6	39	6	17
Other pathologies	5	13	12	20	14	28
Outdoor access	12	10	8	29	9	24

**Table 2:** Number of cats and dogs parasitized by fleas and ticks in our study, Piedmont region.

	Ticks			Fleas	
Animal Species	*Dermacentor marginatus*	*Ixodes* *ricinus*	*Rhipicephalus sanguineus s.l.*	*Ctenocephalides canis*	*Ctenocephalides felis*
Cats (n = 62)	0	14	3	1	44
Dogs (n = 30)	1	10	7	0	12

## Data Availability

Data are contained within the article.

## References

[1] Morelli S., Diakou A., Di Cesare A., Colombo M., Traversa D. (2021). Canine and Feline Parasitology: Analogies, Differences, and Relevance for Human Health. Clin. Microbiol. Rev..

[2] Otranto D., Dantas-Torres F. (2010). Canine and feline vector-borne diseases in Italy: Current situation and perspectives. Parasites Vectors.

[3] Álvarez-Fernández A., Breitschwerdt E.B., Solano-Gallego L. (2018). *Bartonella* infections in cats and dogs including zoonotic aspects. Parasites Vectors.

[4] Deng H., Le Rhun D., Buffet J.-P.R., Cotté V., Read A., Birtles R.J., Vayssier-Taussat M. (2012). Strategies of exploitation of mammalian reservoirs by *Bartonella* species. Vet. Res..

[5] Ruiz J. (2022). JMM Profile: *Bartonella bacilliformis*: A forgotten killer. J. Med. Microbiol..

[6] Ohl M.E., Spach D.H. (2000). *Bartonella quintana* and urban trench fever. Clin. Infect. Dis..

[7] Windsor J.J. (2001). Cat-Scratch Disease: Epidemiology, Etiology, and Treatment. Br. J. Biomed. Sci..

[8] Pennisi M.G., Marsilio F., Hartmann K., Lloret A., Addie D., Belák S., Boucraut-Baralon C., Egberink H.F., Frymus T., Gruffydd-Jones T. (2013). *Bartonella* species infection in cats: ABCD guidelines on prevention and management. J. Feline Med. Surg..

[9] Breitschwerdt E.B. (2008). Feline bartonellosis and cat scratch disease. Vet. Immunol. Immunopathol..

[10] Chomel B.B., Kasten R.W., Henn J.B., Molia S. (2006). *Bartonella* Infection in Domestic Cats and Wild Felids. Ann. N. Y. Acad. Sci..

[11] Fabbi M., Vicari N., Tranquillo M., Pozzi C., Prati P., De Meneghi D., Bertoletti I., Lauzi S., Guiso P., Genchi C. (2004). Prevalence of *Bartonella henselae* in stray and domestic cats in different Italian areas: Evaluation of the potential risk of transmission of Bartonella to humans. Parassitologia.

[12] Zarea A.A.K., Tempesta M., Odigie A.E., Mrenoshki D., Fanelli A., Martella V., Decaro N., Greco G. (2023). The Global Molecular Prevalence of *Bartonella* spp. in Cats and Dogs: A Systematic Review and Meta-Analysis. Transbound. Emerg. Dis..

[13] Ciceroni L., Pinto A., Ciarrocchi S., Ciervo A. (2009). *Bartonella* infections in Italy. Clin. Microbiol. Infect..

[14] Roux V., Eykyn S.J., Wyllie S., Raoult D. (2000). *Bartonella vinsonii subsp. berkhoffii* as an agent of a febrile blood culture-negative endocarditis in a human. J. Clin. Microbiol..

[15] Greene C.E. (2012). Infectious Diseases of the Dog and Cat.

[16] Santilli R.A., Battaia S., Perego M., Tursi M., Grego E., Marzufero C., Gianella P. (2017). *Bartonella*-associated inflammatory cardiomyopathy in a dog. J. Vet. Cardiol..

[17] Lashnits E., Neupane P., Bradley J.M., Richardson T., Thomas R., Linder K.E., Breen M., Maggi R.G., Breitschwerdt E.B. (2020). Molecular prevalence of *Bartonella*, *Babesia*, and hemotropic *Mycoplasma* species in dogs with hemangiosarcoma from across the United States. PLoS ONE.

[18] Iannino F., Salucci S., Di Provvido A., Paolini A., Ruggieri E. (2018). *Bartonella* infections in humans, dogs and cats. Vet. Ital..

[19] Mosepele M., Mazo D., Cohn J. (2011). *Bartonella* Infection in Immunocompromised Hosts: Immunology of Vascular Infection and Vasoproliferation. J. Immunol. Res..

[20] Claasens S., Schwartz I.S., Jordaan H.F., Schneider J.W. (2016). Bacillary angiomatosis presenting with polymorphic skin lesions. IDCases.

[21] Breitschwerdt E.B., Kordick D.L. (2000). *Bartonella* infection in animals: Carriership, reservoir potential, pathogenicity, and zoonotic potential for human infection. Clin. Microbiol. Rev..

[22] Kosoy M., Murray M., Gilmore R.D., Bai Y., Gage K.L. (2003). *Bartonella* Strains from Ground Squirrels Are Identical to *Bartonella washoensis* Isolated from a Human Patient. J. Clin. Microbiol..

[23] Boulouis H.J., Chang C.C., Henn J.B., Kasten R.W., Chomel B.B. (2005). Factors associated with the rapid emergence of zoonotic *Bartonella* infections. Vet. Res..

[24] Traver E.C., Saharia K., Luethy P., Amoroso A. (2024). Severe Infective Endocarditis Caused by *Bartonella rochalimae*. Emerg. Infect. Dis..

[25] Bush J.C., Robveille C., Maggi R.G., Breitschwerdt E.B. (2024). Neurobartonelloses: Emerging from obscurity!. Parasites Vectors.

[26] Telford S.R., Wormser G.P. (2010). *Bartonella* spp. transmission by ticks not established. Emerg. Infect. Dis..

[27] Rocklöv J., Dubrow R. (2020). Climate change: An enduring challenge for vector-borne disease prevention and control. Nat. Immunol..

[28] Ebani V.V., Nardoni S., Fognani G., Mugnaini L., Bertelloni F., Rocchigiani G., Papini R.A., Stefani F., Mancianti F. (2015). Molecular detection of vector-borne bacteria and protozoa in healthy hunting dogs from Central Italy. Asian Pac. J. Trop. Biomed..

[29] Ebani V.V., Guardone L., Marra F., Altomonte I., Nardoni S., Mancianti F. (2020). Arthropod-Borne Pathogens in Stray Cats from Northern Italy: A Serological and Molecular Survey. Animals.

[30] Grippi F., Galluzzo P., Guercio A., Blanda V., Santangelo F., Sciortino S., Vicari D., Arcuri F., Di Bella S., Torina A. (2021). Serological and Molecular Evidence of *Bartonella henselae* in Stray Cats from Southern Italy. Microorganisms.

[31] Dantas-Torres F. (2010). The biology and ecology of the brown dog tick, *Rhipicephalus sanguineus*. Parasites Vectors.

[32] Viani A., Orusa T., Divari S., Lovisolo S., Zanet S., Orusa R., Borgogno-Mondino E., Bollo E. (2025). Detection of *Bartonella* spp. in foxes’ populations in Piedmont and Aosta Valley (NW Italy) coupling geospatially-based techniques. Front. Vet. Sci..

[33] Olmos M.B., Bostik V. (2021). Climate Change and Human Security—The Proliferation of Vector-Borne Diseases Due to Climate Change. Mil. Med. Sci. Lett..

[34] Linardi P.M., Santos J.L.C. (2012). *Ctenocephalides felis* vs. Ctenocephalides canis (Siphonaptera: Pulicidae): Some Issues in Correctly Identifying These Species. Rev. Bras. Parasitol. Vet..

[35] Estrada-Peña A., Mihalca A.D., Petney T.N. (2017). Ticks of Europe and North Africa: A Guide to Species Identification.

[36] Jensen W.A., Fall M.Z., Rooney J., Kordick D.L., Breitschwerdt E.B. (2000). Rapid identification and differentiation of *Bartonella* species using a single-step PCR assay. J. Clin. Microbiol..

[37] Renesto P., Gouvernet J., Drancourt M., Roux V., Raoult D. (2001). Use of *rpoB* gene analysis for detection and identification of *Bartonella* species. J. Clin. Microbiol..

[38] Altschul S., Madden T.L., Schäffer A.A., Zhang J., Zhang Z., Miller W., Lipman D.J. (1997). Gapped BLAST and PSI-BLAST: A new generation of protein database search programs. Nucleic Acids Res..

[39] Hall T.A. (1999). BioEdit: A user-friendly biological sequence alignment editor and analysis program for Windows 95/98/NT. Nucleic Acids Symp. Ser..

[40] McWilliam H., Li W., Uludag M., Squizzato S., Park Y.M., Buso N., Cowley A.P., Lopez R. (2013). Analysis Tool Web Services from the EMBL-EBI. Nucleic Acids Res..

[41] Ronquist F., Teslenko M., Van Der Mark P., Ayres D.L., Darling A., Höhna S., Larget B., Liu L., Suchard M.A., Huelsenbeck J.P. (2012). MrBayes 3.2: Efficient Bayesian Phylogenetic Inference and Model Choice across a Large Model Space. Syst. Biol..

[42] R Core Team (2024). R: A Language and Environment for Statistical Computing.

[43] Maurelli M.P., Pepe P., Colombo L., Armstrong R., Battisti E., Morgoglione M.E., Counturis D., Rinaldi L., Cringoli G., Ferroglio E. (2018). A national survey of Ixodidae ticks on privately owned dogs in Italy. Parasites Vectors.

[44] Giudice E., Di Pietro S., Alaimo A., Blanda V., Lelli R., Francaviglia F., Caracappa S., Torina A. (2014). A molecular survey of *Rickettsia felis* in fleas from cats and dogs in Sicily (Southern Italy). PLoS ONE.

[45] Otranto D., Napoli E., Latrofa M.S., Annoscia G., Tarallo V.D., Greco G., Lorusso E., Gulotta L., Falsone L., Basano F.S. (2017). Feline and canine leishmaniosis and other vector-borne diseases in the Aeolian Islands: Pathogen and vector circulation in a confined environment. Vet. Parasitol..

[46] Sykes J.E. (2022). Greene’s Infectious Diseases of the Dog and Cat.

[47] Bitam I., Dittmar K., Parola P., Whiting M.F., Raoult D. (2010). Fleas and flea-borne diseases. Int. J. Infect. Dis..

[48] Farkas R., Gyurkovszky M., Solymosi N., Beugnet F. (2009). Prevalence of flea infestation in dogs and cats in Hungary combined with a survey of owner awareness. Med. Vet. Entomol..

[49] Persichetti M.F., Solano-Gallego L., Serrano L., Altet L., Reale S., Masucci M., Pennisi M.G. (2016). Detection of vector-borne pathogens in cats and their ectoparasites in southern Italy. Parasites Vectors.

[50] Morelli S., Crisi P.E., Di Cesare A., De Santis F., Barlaam A., Santoprete G., Parrinello C., Palermo S., Mancini P., Traversa D. (2019). Exposure of client-owned cats to zoonotic vector-borne pathogens: Clinic-pathological alterations and infection risk analysis. Comp. Immunol. Microbiol. Infect. Dis..

[51] Chang C.C., Chomel B.B., Kasten R.W., Romano V., Tietze N. (2001). Molecular evidence of *Bartonella* spp. in questing adult *Ixodes pacificus* ticks in California. J. Clin. Microbiol..

[52] Duplan F., Davies S., Filler S., Abdullah S., Keyte S., Newbury H., Morin-Adeline V., Helps C., Tasker S., Tay S. (2018). *Anaplasma phagocytophilum*, *Bartonella* spp., haemoplasma species and *Hepatozoon* spp. in ticks infesting cats: A large-scale survey. Parasites Vectors.

[53] Eskow E., Rao R.V., Mordechai E. (2001). Concurrent infection of the central nervous system by *Borrelia burgdorferi* and *Bartonella henselae*: Evidence for a novel tick-borne disease complex. Arch. Neurol..

[54] Wikswo M.E., Hu R., Metzger M.E., Eremeeva M.E. (2007). Detection of *Rickettsia rickettsii* and *Bartonella henselae* in *Rhipicephalus sanguineus* ticks from California. J. Med. Entomol..

[55] Regier Y., Ballhorn W., Kempf V.A.J. (2017). Molecular detection of *Bartonella henselae* in 11 *Ixodes ricinus* ticks extracted from a single cat. Parasites Vectors.

[56] Stensvold C.R., Marai D., Andersen L.O., Krogfelt K.A., Jensen J.S., Larsen K.S., Nielsen H.V. (2015). *Babesia* spp. and other patho-gens in ticks recovered from domestic dogs in Denmark. Parasites Vectors.

[57] Dietrich F., Schmidgen T., Maggi R.G., Richter D., Matuschka F.R., Vonthein R., Breitschwerdt E.B., Kempf V.A. (2010). Prevalence of *Bartonella henselae* and *Borrelia burgdorferi* sensu lato DNA in ixodes ricinus ticks in Europe. Appl. Environ. Microbiol..

[58] Halos L., Jamal T., Maillard R., Beugnet F., Le Menach A., Boulouis H.J., Vayssier-Taussat M. (2005). Evidence of *Bartonella* sp. in questing adult and nymphal *Ixodes ricinus* ticks from France and co-infection with *Borrelia burgdorferi* sensu lato and *Babesia* sp.. Vet. Res..

[59] Sykes J.E., Lindsay L.L., Maggi R.G., Breitschwerdt E.B. (2010). Human coinfection with *Bartonella henselae* and two hemotropic mycoplasma variants resembling *Mycoplasma ovis*. J. Clin. Microbiol..

[60] Wechtaisong W., Bonnet S.I., Lien Y.Y., Chuang S.T., Tsai Y.L. (2020). Transmission of *Bartonella henselae* within Rhipicephalus sanguineus: Data on the Potential Vector Role of the Tick. PLOS Neglected Trop. Dis..

[61] Breitschwerdt E.B., Maggi R.G., Duncan A.W., Nicholson W.L., Hegarty B.C., Woods C.W. (2007). *Bartonella* species in blood of immunocompetent persons with animal and arthropod contact. Emerg. Infect. Dis..

[62] Breitschwerdt E.B., Maggi R.G., Nicholson W.L., Cherry N.A., Woods C.W. (2008). *Bartonella* sp. bacteremia in patients with neurological and neurocognitive dysfunction. J. Clin. Microbiol..

[63] Edvinsson M., Norlander C., Nilsson K., Mårtensson A., Skoog E., Olsen B. (2021). *Bartonella* spp. seroprevalence in tick-exposed Swedish patients with persistent symptoms. Parasites Vectors.

[64] Gutiérrez R., Krasnov B., Morick D., Gottlieb Y., Khokhlova I.S., Harrus S. (2015). *Bartonella* infection in rodents and their flea ectoparasites: An overview. Vector Borne Zoonotic Dis..

[65] Brunetti E., Fabbi M., Ferraioli G., Prati P., Filice C., Sassera D., Dalla Valle C., Bandi C., Vicari N., Marone P. (2013). Cat-scratch disease in Northern Italy: Atypical clinical manifestations in humans and prevalence of *Bartonella* infection in cats. Eur. J. Clin. Microbiol. Infect. Dis..

[66] Lapi F., Marconi E., Ferroglio E., Cricelli J., Rossi A., Cricelli C. (2025). The burden of some of the most common zoonoses in primary care: A population-based study in Italy. Postgrad. Med. J..

[67] Garcia-Sanchez P., Romero-Trancón D., Falces-Romero I., Navarro Carrera P., Ruiz-Carrascoso G., Carmena D., Casares Jiménez M., Rivero-Juárez A., Moya L., Rodón J. (2024). Zoonosis screening in Spanish immunocompromised children and their pets. Front. Vet. Sci..

[68] Lantos P.M., Maggi R.G., Ferguson B., Varkey J., Park L.P., Breitschwerdt E.B., Woods C.W. (2014). Detection of *Bartonella* species in the blood of veterinarians and veterinary technicians: A newly recognized occupational hazard?. Vector Borne Zoonotic Dis..

[69] Oteo J.A., Maggi R., Portillo A., Bradley J., García-Álvarez L., San-Martín M., Roura X., Breitschwerdt E. (2017). Prevalence of *Bartonella* spp. by culture, PCR and serology, in veterinary personnel from Spain. Parasites Vectors.

[70] Giannelli A., Schnyder M., Wright I., Charlier J. (2024). Control of companion animal parasites and impact on One Health. One Health.

